# The GlycanBuilder: a fast, intuitive and flexible software tool for building and displaying glycan structures

**DOI:** 10.1186/1751-0473-2-3

**Published:** 2007-08-07

**Authors:** Alessio Ceroni, Anne Dell, Stuart M Haslam

**Affiliations:** 1 Division of Molecular Biosciences, Imperial College London, SW7 2AZ, UK

## Abstract

**Background:**

Carbohydrates play a critical role in human diseases and their potential utility as biomarkers for pathological conditions is a major driver for characterization of the glycome. However, the additional complexity of glycans compared to proteins and nucleic acids has slowed the advancement of glycomics in comparison to genomics and proteomics. The branched nature of carbohydrates, the great diversity of their constituents and the numerous alternative symbolic notations, make the input and display of glycans not as straightforward as for example the amino-acid sequence of a protein. Every glycoinformatic tool providing a user interface would benefit from a fast, intuitive, appealing mechanism for input and output of glycan structures in a computer readable format.

**Results:**

A software tool for building and displaying glycan structures using a chosen symbolic notation is described here. The "GlycanBuilder" uses an automatic rendering algorithm to draw the saccharide symbols and to place them on the drawing board. The information about the symbolic notation is derived from a configurable graphical model as a set of rules governing the aspect and placement of residues and linkages. The algorithm is able to represent a structure using only few traversals of the tree and is inherently fast. The tool uses an XML format for import and export of encoded structures.

**Conclusion:**

The rendering algorithm described here is able to produce high-quality representations of glycan structures in a chosen symbolic notation. The automated rendering process enables the "GlycanBuilder" to be used both as a user-independent component for displaying glycans and as an easy-to-use drawing tool. The "GlycanBuilder" can be integrated in web pages as a Java applet for the visual editing of glycans. The same component is available as a web service to render an encoded structure into a graphical format. Finally, the "GlycanBuilder" can be integrated into other applications to create intuitive and appealing user interfaces: an example is the "GlycoWorkbench", a software tool for assisted annotation of glycan mass spectra. The "GlycanBuilder" represent a flexible, reliable and efficient solution to the problem of input and output of glycan structures in any glycomic tool or database.

## Background

Carbohydrates are the most abundant type of biological molecules. The smallest structural unit of a carbohydrate is called a *monosaccharide*. Chemically, monosaccharides are aldehydes or ketones with two or more hydroxyl groups. They can exist as linear molecules but more usually they cyclize to form ring structures. Complex carbohydrates (also referred to as *glycans*) are formed by combinations of monosaccharides covalently linked by glycosidic bonds: two types of glycosidic bonds can be formed, α and β, depending on the orientation of the anomeric centres of the monosaccharides involved. Each hydroxyl group of a monosaccharide constitutes a possible point of formation for a glycosidic bond. Therefore, glycans can have very complex structures with many branching points. The monosaccharides are classified according to the number of carbon atoms they contain, the position of the anomeric centre, and its chiral handedness. Glycans are classified accordingly to the number of monosaccharide units they contain: large polysaccharides (such as cellulose, starch and chitin) containing many thousand repeating units whereas smaller oligosaccharides can contain between two and ten monosaccharides.

Carbohydrates can be found as homo-polymers but are usually attached to other biomolecules such as lipids or proteins to form complexes named *glycoconjugates*. Apart from their well known use in energy storage and expenditure, the roles of carbohydrates in living organisms are varied and fundamental. Glycans can have structural and modulatory functions by themselves or can modulate the function of the molecules to which they are attached by the specific recognition of the glycan structure by carbohydrate-binding proteins (lectins). Glycans regulate both the folding and degradation of proteins. Moreover, since the outer cell membrane is covered by carbohydrates, they mediate interactions with other cells of the same organism or other pathogenic organisms such as viruses, bacteria and multi-cellular parasites. The critical role of glycans in diseases and their utility as biomarkers for pathological conditions have now been widely recognized [1] and have fostered major interest towards the characterization of the glycome, the entire repertoire of glycans expressed in a cell, tissue or whole organism.

### Glycoinformatics

The additional complexity of glycan structures compared to proteins and nucleic acids has slowed the advancement of glycomics. The branched structure of glycans means that most of the bioinformatic tools developed for analysis of linear proteins and nucleic acids cannot be simply adapted to the sequencing of glycan structures. Moreover, no extensive database of known glycan structures and related primary data is currently available, although various initiatives in this direction are currently in place. Therefore, the current situation sees a critical lack of software tools for almost every aspect of glycomic research [2].

The branched non-sequential nature of glycan structures and the great diversity of their constituents imply that the input of a structure into a computer readable format is not as straightforward as writing a sequence of characters for representing the amino acid sequence of a protein chain. Additionally, numerous alternative symbolic notations are commonly adopted to represent glycan structures in publications. Every glycoinformatic software tool providing a user interface would benefit from a fast, intuitive, graphically appealing mechanism for input and output of glycan structures. For example, database application would profit from an easy to use interface for performing structure searches and displaying search results.

A user friendly input/output tool for glycan structures should satisfy three fundamental requirements: provide an intuitive interface to build structures with minimal user interaction, allow the encoding in a standard format also readable with other software tools and create conventional and appealing graphical representations of glycans. These requirements are usually diverging. A common practice is to employ graphical editors for drawing the structures to be published in research papers. Graphical editors give the user the highest freedom in creating the graphical representation. However, it is realistically impossible for a software tool to extract useful information about the glycan structure from the resulting geometrical representation. Moreover, graphical editors require a large amount of user interaction for conveying the desired result. Some input tools that have been developed for searching structure databases mimic the operations of a graphical editor. The symbols for saccharides and linkages are usually predetermined but the user can freely position the saccharides on the drawing board. Two editors follow this approach: KCAM [3,4] and DrawRINGS [5]. Free positioning of residues implies a much easier task for the tool, but it still requires intensive interaction for the user. It is common that interfaces of structure databases are written in HTML and are thus constrained by the limits of this language. The structure is defined by choosing between a limited set of templates and they are more focused on usability than visual appeal. Several examples can be found: the glycan database of the Consortium for Functional Glycomics [6], the Bacterial Carbohydrate Structure Data Base [7] and the Glycosciences.de structure database [8].

The EUROCarbDB design studies [9] aim to create the foundations for databases and bioinformatic tools in the realm of glycobiology and glycomics. EUROCarbDB will establish the technical framework for bottom to top initiatives where all interested research groups can feed in their primary data. The importance of the EUROCarbDB initiative in the development of glycan structure databases has been widely recognized [1,2]. The "GlycanBuilder" has been developed for the EUROCarbDB project with the purpose of creating a fast, intuitive and flexible software tool for building and displaying glycan structures. The "GlycanBuilder" will constitute the main interface for structure searches and the display of the results in the EUROCarbDB databases. The tool uses an automatic rendering algorithm to represent the glycans in the chosen symbolic notation. The rendering algorithm derives the information about the symbolic notation from a configurable graphical model as a set of rules determining the style and placement of residues and linkages. The automatic rendering algorithm enables the "GlycanBuilder" to be used for input of structures with minimal user intervention or as a component for automated output of high-quality compact representations of glycans.

### Structure Encoding

The representation of glycan structures in a computer readable format is an essential prerequisite for the exchange of information between databases and for the use of stored data by bioinformatic tools. The string encoding format for glycan structures proposed by IUPAC-IUBMB [10] was explicitly designed to be easily written and understood by researchers. A problem with the IUPAC format is the possibility of representing the same structure in different ways by varying the order of its branches. This issue was addressed in the SWEET-DB database [11] using the LINUCS format [12]. A later encoding format with unique structure representation, GLYDE [13], has been introduced to facilitate parsing from software tools by using XML to represent glycan structures.

A critical problem with the previous formats is exemplified by the representation of monosaccharide components. The convention used for naming monosaccharide monomers results in multiple names for the same basic chemical configuration. This ambiguity is not easily addressed by software tools. One of the efforts taken by the EUROCarbDB project has been the development of a controlled vocabulary for monosaccharides centred on a database of monosaccharides units. Each unit is represented as an unmodified saccharide base type (e.g. glucose) plus a list of substituents and modifications. EUROCarbDB has proposed a glycan encoding format based on this monosaccharide representation, Glyco-CT [14]. Glyco-CT describes a glycan structure in XML as a set of base type saccharides and substituents, and a set of connections. This format will be used by EUROCarbDB to store structures in the database. The Glyco-CT monosaccharide naming scheme will also be adopted in the second version of GLYDE [15], recently recognized by glycomic experts as the much needed standard format for exchange of glycan structures [2].

The "GlycanBuilder" uses the Glyco-CT format for the import/export of glycan structures, while suitable libraries are being developed by EUROCarbDB for translating Glyco-CT into other common encoding formats and vice versa.

### Symbolic Notations

Symbolic representations of glycans consist of a series of geometric symbols that represent monosaccharide units connected by lines to indicate glycosidic linkages. Symbolic notations are widely used in publications, especially for representing mammalian structures, because they enable a very compact way of describing complex glycans, useful when a large series of structures needs to be displayed (i.e. the profile of glycans expressed by a cell population). Moreover, symbolic representations are much easier for humans to recognize and allow a rapid comparison of structures when determining differential expressions in biological contexts. Unfortunately, no standard notation exists and many different types of symbols and conventions can be found in the literature.

**Figure 1:**
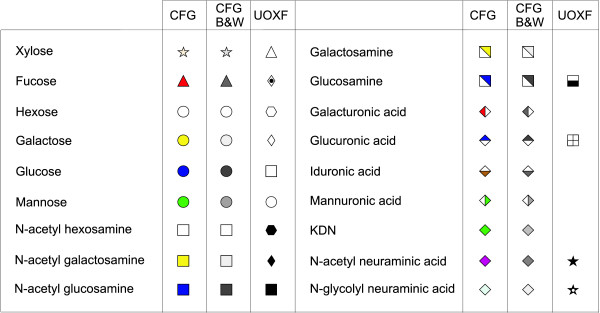
**Symbolic representation of monosaccharides**. Representation of monosaccharides with geometric shapes as described in the notations used by the Consortium for Functional Glycomics (CFG) and the Oxford Glycobiology Institute (UOXF).

The Consortium for Functional Glycomics (CFG) has issued a recommendation for the symbolic representation of the glycan structures present in mammalian organisms. This notation is used by all Consortium databases and is being employed in the second edition of the book "Essentials of Glycobiology" [16]. Due to the popularity of the book and the scientific impact of the Consortium resources, this notation has been widely accepted, as exemplified by its utilization by other major international glycobiology initiatives such as HUPO Human Disease Glycomics/Proteome Initiative based in Japan [17], and is likely to become the standard. However, since this notation has been devised for a specific category of biological systems, the representation of the saccharides that are not found in mammalian glycans remains unspecified. The saccharides are represented by coloured geometric shapes (Figure 1): the same shape indicates same monosaccharide super class (e.g. Hexose, Deoxy-Hexose) while the same colouring indicates same stereochemistry (e.g. galactose, galactosamine, N-acetyl galactosamine). A black and white coded version of these symbols is also available (Figure 1). Anomeric and linkage information is displayed as text in the proximity of the line representing the glycosidic bond. Modifications of monosaccharides are represented as text attached to the saccharide symbol.

**Figure 2:**
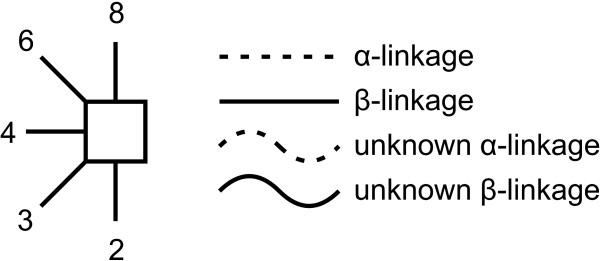
**Orientation and style of linkages in UOXF notation**. In the symbolic notation adopted by the Oxford Glycobiology Institute the anomeric state is represented by varying the style of the line (dashed or continuous) while the linkage position is represented by the orientation of the saccharide in respect to its parent.

**Figure 3:**
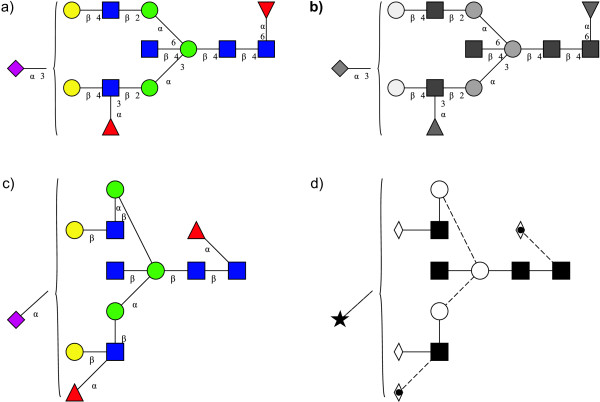
**Example of a structure represented using CFG and UOXF notations**. The same structure is drawn here using four different symbolic notations: a) the CFG standard notation, b) the CFG black and white notation, c) the CFG hybrid notation with placement of residues in respect to linkage position, and d) the UOXF notation. These drawings have been created using the "GlycanBuilder".

A second proposal for representing linkage information, originally suggested in [18], has been adopted by the Oxford Glycobiology Institute (UOXF) and is detailed in [19]. In this notation, the anomeric state is represented by varying the style of the line (dashed or continuous) while the linkage position is visually represented by the orientation of the saccharide in respect of its parent (Figure 2). A curved line is used for the linkage if the position is unknown. The symbols used for saccharides are all in black and white and are limited when compared to those used by the CFG (Figure 1). A mixed notation using CFG symbols and UOXF linkage notation has also been proposed. Example of a structure depicted with CFG and UOXF notations is given in Figure 3.

## Implementation

The "GlycanBuilder" is based on an automatic rendering algorithm able to transform a computer encoded glycan structure into a pictorial representation determined by the chosen symbolic notation. The encoded structure is first parsed and transformed into a data object that stores all the information about the glycan (saccharides, substituents, modifications, linkages, markers ...). The data object is then rendered into the desired output media using a graphical model representing the symbolic notation of choice. The rendering process is completely automatic: the spatial placement and aspect of residues depends only on the symbolic notation. This means the "GlycanBuilder" can be used as an automated component for displaying glycan structures. Additionally, when the "GlycanBuilder" is employed as an interactive drawing tool, this feature makes the user free from any responsibility regarding the drawing and creates faster and easier-to-use interfaces.

The internal object used to store and manage information about glycans in the "GlycanBuilder" is a tree structure whose nodes are the glycan residues and whose edges are their linkages. Each node represents a drawable object, an enlarged concept of structural constituent comprising: saccharides, reducing-end specificators and markers, substituents and saccharide modifications. Reducing-end specificators are used to identify possible modifications at the reducing-end terminal (or no modifications). A special node is also defined for collecting glycan terminals with unspecified linkages, a common way of describing structures with incomplete topological information (like the structure in Figure 3). This "bracket" node has no parent and all the uncertain terminals are stored as its children.

The symbolic notation is represented in the internal graphical model as a set of rules governing the position of a residue around its parent and the drawing style of residues and linkages. These rules are used by the rendering algorithm to decide how to graphically represent the nodes and edges of the internal data object. The graphical model is parsed from a configuration file that can easily be updated with new notations. The graphical model is a separate entity from the glycan renderer and can readily be exchanged to pass from one notation to another.

The output of the rendering process can be redirected to a graphical component to create a user interface. The user can thus interact with the displayed structure and ask for modifications of the glycan object, which is then redisplayed. Alternatively, the output can be transformed into a bitmapped or a vector graphic image, useful for showing the results of a database search or for publication purposes.

The glycan rendering process is composed of three phases. Firstly, each residue is positioned in a generic spatial region around its parent node. Secondly, the bounding box of each residue is computed as to optimize the spatial occupation of the displayed structure and to prevent collisions between nodes and edges. Finally, the symbols for residues and linkages are displayed using the coordinates provided by the bounding boxes. Each phase is performed using recursive algorithms that navigate the tree structure of the glycan.

### Positioning

**Figure 4:**
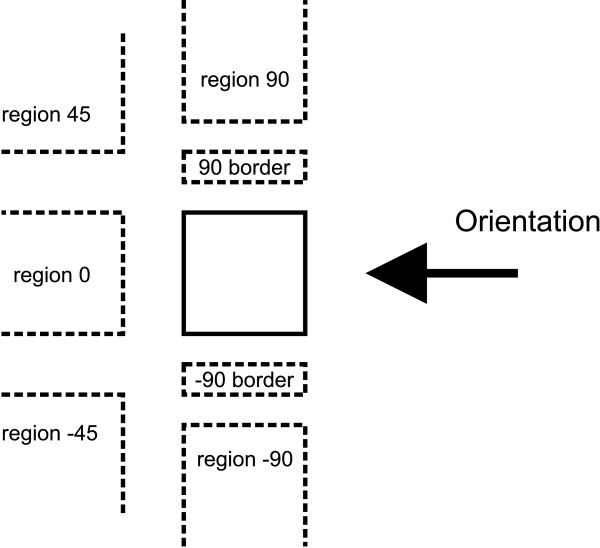
**Available positions for a residue around its parent**. During the positioning phase of the rendering algorithm each residue is placed in a generic spatial region around its parent. These regions are used during the computation of bounding boxes to decide the alignment and spatial placement of each residue's children. The position is identified by the relative angle at which the region is located with respect to the orientation of the parent.

During the positioning phase each residue is placed in a generic spatial region around its parent (Figure 4). These regions are used during the computation of bounding boxes to decide the alignment and spatial placement of each residue's children. A position is identified by the relative angle at which the region is located with respect to the orientation of the parent. The orientation of each residue is initially the same as the global orientation of the structure (right-to-left, left-to-right, bottom-to-top, or top-to-bottom) but can be changed at each step accordingly with the selected position. Two of the available positions are referred to as "border" regions: border positions have special treatments, occupy less space and are drawn only as text; border positions are used for displaying substituents and modifications.

**Figure 5:**
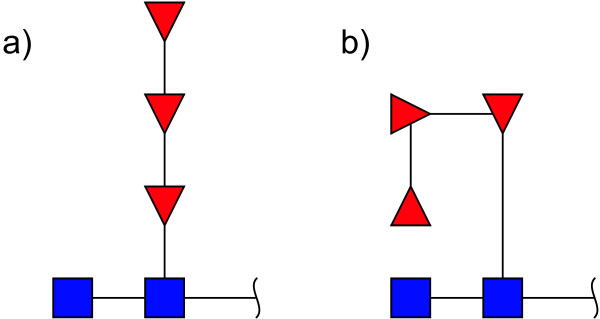
**Effect of the "sticky" attribute**. This figure shows the effect of the "sticky" attribute on the placement of residues. If a position is "sticky", all the subsequent children are placed in position 0: in this way it is possible to force sub-trees in regions with a change in orientation to be drawn as a sequences (a), thus avoiding the creation of spiralling series of residues (b). The structure drawn in this figure does not represent an actual glycan.

The positioning algorithm navigates recursively from the root to the leaves of the tree structure using the set of rules in the graphical model to decide the positions of the children of each node. A positioning rule is composed by a matching operator and a set of attributes. The matching operator identifies if a rule applies to a triplet comprised by the parent residue, the child residue and the linkage. The matching operator is a Boolean function constituted of logical conditions combined using Boolean operators (and, or, negation, parenthesis). Each condition matches a certain parent, linkage or child attribute: residue type, linkage position, anomeric state, position of the anomeric carbon or residue class (e.g. saccharides, reducing ends, brackets ...). The attributes of a rule are: a set of possible positions for this residue, a flag identifying if the residue is on a border region, a flag identifying if the orientation of the child is the same as the parent or should rotate accordingly to the position angle, a flag identifying if the position is "sticky". If a position is "sticky", all the subsequent children are placed in position 0: in this way it is possible to force sub-trees in regions with a change in orientation to be drawn as a sequences, thus avoiding the creation of spiralling series of residues (Figure 5). Examples of positioning rules are:

If linkagePosition==6 then // *UOXF*

      position=45;

      border=false;

      change orientation = false;

      sticky=false;

If residueType==Fucose then // *CFG*

      position={-90,90};

      border=false;

      change orientation = true;

      sticky=true;

If residueClass==Substituent then // *Both*

      position={-90,90};

      border=true;

      change orientation = false;

      sticky=false;

After the rules have been applied, the positioning algorithm has to decide the actual position of each node among the possible choices. All the children of a residue are considered altogether in order to better redistribute them in the space around the parent. The placement of each node is decided starting from the residues with stricter constraints. Firstly, all the residues with a single possible position are assigned. Secondly, the border residues are sequentially placed in an empty border position if available or in the less crowded position otherwise. When deciding the less crowded position the booking algorithm makes no difference between border and non-border regions. Finally, the remaining children are sequentially placed in the less crowded positions. The automatic placement can be overridden on user request, in case a specific arrangement of the structure is needed to represent particular conditions.

### Computation of Bounding Boxes

**Figure 6:**
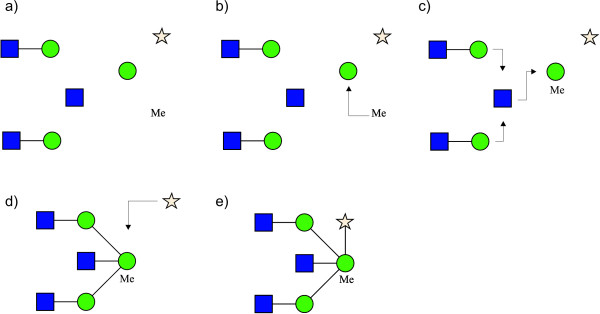
**Phases of the rendering algorithm, right-to-left orientation**. The algorithm for computing residue bounding boxes is the core component of the rendering process. The bounding box of each residue is computed to optimize the spatial occupation of the displayed structure and to prevent collisions between nodes and edges. The algorithm recursively navigates through the tree structure performing the following phases at each step: a) compute the bounding boxes of the current residue and its sub-trees by grouping for region, b) align border regions with the current residue and place them on bottom and/or top, c) align left regions (-45, 0, 45) and place them on the left of the current residue without clashing with the border regions, d) align regions (-90, 90) with current residue and place them on bottom and/or top of the residue not clashing with the other regions, e) display results. The structure drawn in this figure does not represent an actual glycan or part of it.

The algorithm for computing the bounding boxes of residues is the core component of the rendering process. The bounding box indicates at which coordinates a residue will be displayed on the output media. The procedure is described here only for a right-to-left orientation: the others are simply rotated by multiples of 90 degrees with respect to this (diagonal orientations are not allowed). The algorithm recursively navigates through the tree structure performing the following phases for each node (Figure 6):

1. compute the bounding box of the current residue with a free spatial placement (Figure 6a);

2. recursively compute the bounding boxes for all the sub-trees by grouping for region and align the sub-trees in each region (Figure 6a);

3. align centre of border regions -90 and 90 with the current residue and place the regions on the bottom and top of the residue (Figure 6b);

4. align left regions (-45, 0, 45) and place them on the left of the current residue without clashing with border regions (Figure 6c);

5. align centre of regions -90 and 90 with current residue and place the regions on the bottom and top of the residue not clashing with the other regions (Figure 6d).

**Figure 7:**
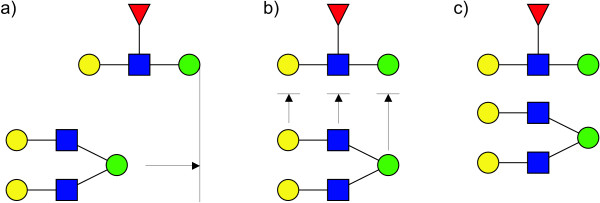
**Alignment and collision solving**. Example of alignment between two sub-trees: a) align the roots of the two sub-trees on the right of their bounding boxes; b) compute the size of the shift needed to move one sub-tree on the bottom of the other maintaining a minimum distance between all their nodes; c) translate all the residues of the sub-tree according to the computed shift value.

During phase 2 each region is computed separately. Firstly, the bounding boxes are computed recursively for each residue of every sub-tree. Once the bounding boxes of the residues of a sub-tree are computed, they are aligned with the residues of the adjacent sub-tree in the region. The alignment of two sub-trees is computed clockwise (i.e. those in region 0 are aligned top-to-bottom, those in region -90 are aligned left-to-right ...). The alignment is performed to minimize the spatial distance between the sub-trees and to avoid collisions between their residues. An example of top-to-bottom alignment of one sub-tree on the right of another is given in Figure 7:

1. align the roots of the two sub-trees on the right of their bounding boxes (Figure 7a);

2. compute the size of the shift needed to move one sub-tree on the bottom of the other maintaining a minimum distance between all their nodes (Figure 7b);

3. translate all the residues of the sub-tree according to the computed shift value (Figure 7c).

This alignment and collision solving algorithm is used during all the other phases to align entire regions. The alignment and collision solving algorithm is the most delicate part of the rendering process and it is responsible for the optimal placement of residues.

**Figure 8:**
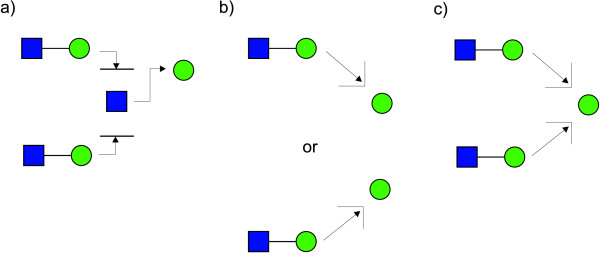
**Alignment of region -45, 0 and 45 with current residue, right-to-left orientation**. a) If region 0 is not empty, region -45 and region 45 are aligned at right with region 0 and placed on the bottom and the top of it respectively. After that, the centre of region 0 is aligned with the centre of the current residue and the three regions are placed on the left of current residue. b) If region 0 is empty and only one region between -45 and 45 contains residues, the non-empty region is placed on the corner of the current residue, on top-left or bottom-left accordingly. c) If region 0 is empty, the regions -45 and 45 are placed on the corners of the current residue (top-left or bottom-left accordingly).

The alignment of left regions (-45, 0, 45) with the current residue has three special cases (Figure 8). If region 0 is not empty, region -45 and region 45 are aligned at right with region 0 and placed on the bottom and the top of it respectively (Figure 8a). After that, the centre of region 0 is aligned with the centre of the current residue and the three regions are placed on the left of current residue. If region 0 is empty and only one region between -45 and 45 contains residues, the non-empty region is placed on the corner of the current residue, on top-left or bottom-left accordingly (Figure 8b). Finally, if both region -45 and 45 are non empty, they are first aligned on their rights and then placed symmetrically on the left of current residue (Figure 8c).

The computation of bounding boxes for the bracket residue and its children (antennae with uncertain linkage) is performed separately. The bounding boxes for these residues are computed after the rest of the structure. The bracket residue is placed on the left of the structure and its bounding box is as large as the whole structure. Its children are aligned top to bottom on the right of the bracket. For each antenna the bounding boxes are computed as for the normal structure.

### Drawing

Residues and linkages are drawn as geometrical shapes and not as bitmaps so that resolution is not affected at different sizes and scales. A residue is drawn by fitting the chosen shape inside the bounding box, while a linkage is displayed by drawing a styled line connecting the centre of the parent's bounding box with that of the child. The aspect of residues and linkages is part of the graphical model and it is also stored in configuration files. The residue style matches a single residue type. Various graphical attributes can be set for the residue style: shape (triangle, circle, square, rhombus, diamond, hexagon, heptagon, and special shapes for reducing-end and bracket), fill colour (RGB format), fill style (empty, full, half full, chequered), associated text and text colour. Some of the shapes, like those for reducing-ends, are oriented to point towards either the parent or the children residue. The linkage style is decided using the same matching operator described for positioning rules. The graphical attributes that can be set for a linkage are: line style (dashed, continuous), line shape (empty, straight, curved) and which linkage information to display. Anomeric state and linkage position can be displayed: never, always, only if known. Linkage information is displayed near the linkage line, next to the corresponding residue: anomeric state is displayed next to the child and linkage position next to the parent.

## Results and Discussion

**Figure 9:**
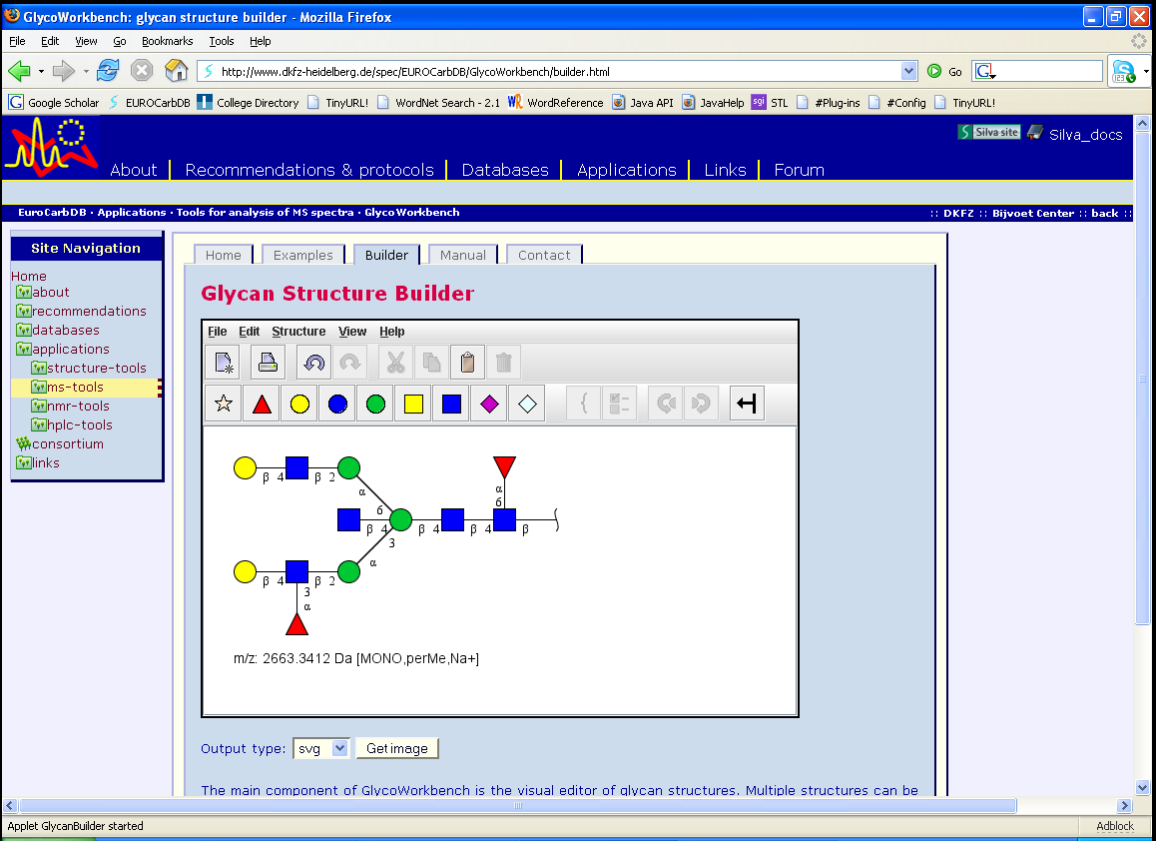
**EUROCarbDB page of the "GlycanBuilder" applet**. The "GlycanBuilder" Java applet is a visual editor for glycan structures that can be integrated in standard web pages. The applet is part of the forthcoming web interface of the EUROCarbDB database and is currently available for testing from the EUROCarbDB homepage [23].

The automatic rendering algorithm detailed in the previous section enable the "GlycanBuilder" to be employed in several different applications. A visual editor for glycan structures has been developed both as a stand-alone Java application [see additional file 1] and a Java applet that can be integrated in standard web pages. The applet is part of the forthcoming web interface of the EUROCarbDB database and is currently available for testing from the EUROCarbDB homepage (Figure 9). The user creates a structure in the editor by sequentially adding monosaccharides, modifications or reducing-end markers. Each addition is done by selecting the point of attachment and performing the desired action. The placement is decided by the rendering algorithm on the basis of the symbolic notation as described above. The user can change the symbolic notation by selecting from: CFG (normal B&W and with linkage placement), UOXF and 2D text. The available structural constituents comprise a comprehensive and continuously updated list of saccharides, substituents, reducing-end markers and saccharide modifications. A library of common structural motifs (core and terminals) is included for convenience. All the stereo-chemical information about a saccharide, like anomeric conformation, chirality, ring configuration and linkage position, can be specified. The usual editing functions are available: cut & copy, undo/redo and drag & drop. Finally, the user can export the structure encoded as a Glyco-CT string or rendered in a standard graphical format (EPS, PDF, SVG, PNG, JPG are supported). The export of structures in graphical format is performed by submitting the encoded structure to a web service developed using the same automatic rendering algorithm. The web service accepts a structure in Glyco-CT format, a set of attributes specifying the symbolic notation and the desired output format. The output of the web service is a file representing the rendered structure in the chosen format. The web service is hosted on the EUROCarbDB server and uses the standard HTTP protocol for input and output. The GlycanBuilder is also available as a Java library that can be integrated in other applications and bioinformatic tools for managing the input and output of glycan structures.

### GlycoWorkbench: Assisted Annotation of Glycan Fragment Mass Spectra

**Figure 10:**
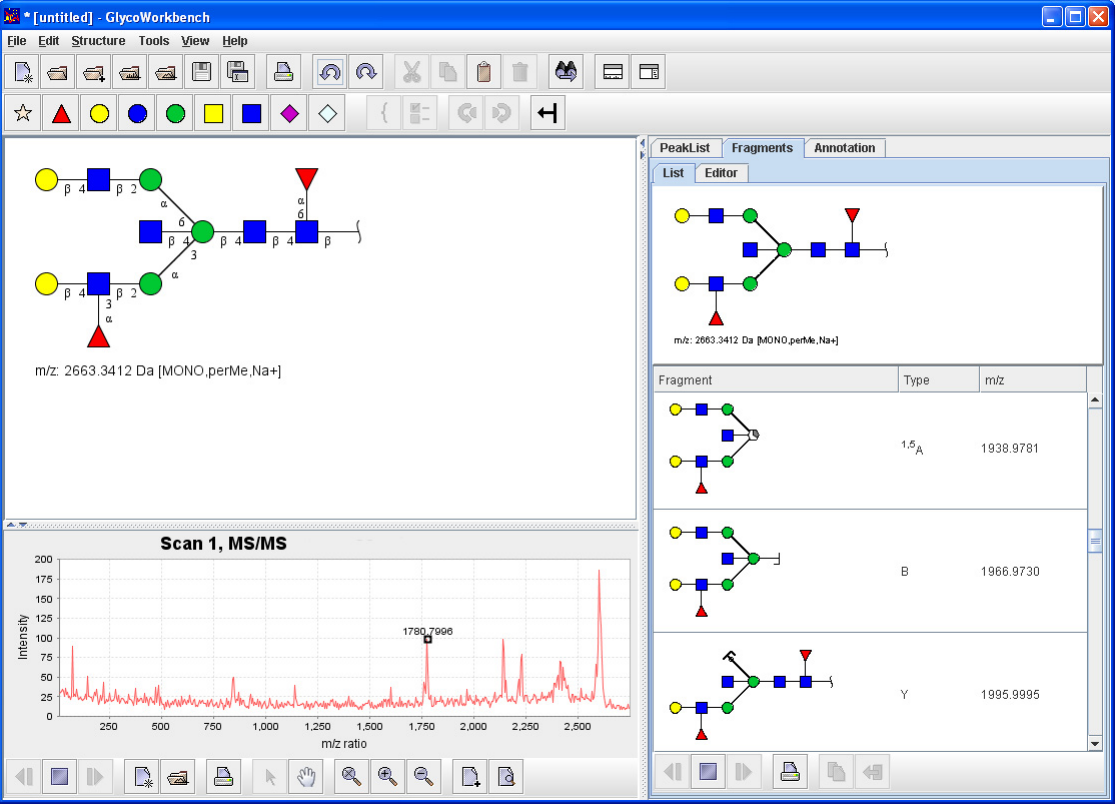
**"GlycoWorkbench"**. The "GlycoWorkbench" is an integrated suite of software tools designed for assisting the annotation of glycan fragment mass spectra during glycan sequencing. The "GlycoWorkbench" uses the "GlycanBuilder" as an internal structure editor and as a component to display structures and fragments in its various views. The application can be freely downloaded and installed from the EUROCarbDB homepage [22].

Mass spectrometry is the main analytical technique currently used to address the challenges of glycomics [20] as it offers unrivalled levels of sensitivity and the ability to handle the complex mixtures of different glycan variations. Modern MS techniques are capable of producing mass spectra of fragmented carbohydrates and this information can be exploited to resolve the structure of a glycan molecule [21]. The "GlycanBuilder" has been used to create the user interface of a software tool for assisting the interpretation of mass spectra of glycans, the "GlycoWorkbench" (Figure 10). The "GlycoWorkbench" lets the user build potential glycan structures, compute their fragments, load and display raw spectra files, create or load peak lists and automatically assign fragment ions to mass values. "GlycoWorkbench" uses the "GlycanBuilder" as an internal structure editor and as a component to display structures and fragments. The fragment ions are displayed using special symbols for glycosidic bond cleavages and ring-fragments. The placement of these symbols is decided regarding to the cleaved residue and not the cleavage it-self, in this way the display is consistent between a fragment and the intact structure. The "GlycoWorkbench" is written in Java and can be freely downloaded and installed from the EUROCarbDB homepage [22]. A full description of the software tool will be published soon.

## Conclusion

The "GlycanBuilder", a software tool for building and displaying glycan structures, has been presented here. The tool is based on an automatic rendering algorithm that can draw glycan structures in a chosen symbolic notation with no user intervention. The symbolic notation is encoded in a graphical model as a set of rules specifying the style and placement of structure residues. The graphical model is independent from the rendering algorithm and can readily be exchanged to specify different symbolic notations. The rendering algorithm is able to produce high-quality compact representations of structures which are ready for publication purposes. The type of structures that can be represented is not restricted to any particular type or biological context. The algorithm is inherently fast and is able to draw a structure using only few traversals of the glycan tree, with no iterative optimization steps. The automated rendering process enables the "GlycanBuilder" to be used both as a user-independent component for displaying glycan structures and as a rapid and easy-to-use drawing tool. The computer encoded structures can be imported into the tool and exported from it using the Glyco-CT format. The "GlycanBuilder" can thus be integrated in other applications to create intuitive user interfaces for input of glycans or to display encoded structures using symbolic notations. The tool is available as a stand-alone Java application [see additional file 1], as a Java applet that can be integrated in web pages, as a web service accessible via HTTP to create graphic files from encoded structures and as a library that can be easily added to other software packages. The "GlycanBuilder" represent a flexible, reliable and efficient solution to the problem of input and output of glycan structures in any glycomic tool or database.

## Availability and Requirements

• Project name: EuroCarbDB – GlycanBuilder

• Project home page: 

• Operating system(s): Platform independent

• Programming language: Java

• Other requirements: Java 5.0 or higher

• License: GNU GPL

• Any restrictions to use by non-academics: no licence needed

## Competing interests

The author(s) declare that they have no competing interests.

## Authors' contributions

AC developed the software tool and tested it, created the website and drafted the manuscript. AD oversaw the project, recognized the validity of the approach, and edited the paper. SH participated in the definition of the requirements of the software, tested the software tool and helped to draft the manuscript. All authors read and approved the final manuscript.

## Supplementary Material

Additional file 1 The ZIP archive contains the stand-alone version of the "GlycanBuilder" structure editor. To install the tool, extract the content of the ZIP archive in a folder of your choice. To run the tool, double click on the file GlycanBuilder.jar. The tool has been tested under Windows, Linux and Mac OS X. In order to run the GlycanBuilder tool, the Java Runtime Environment (JRE) version 5.0 must be installed on the computer. Click here for file
